# Effect of different post-partum therapeutic protocols with intrauterine oxytetracycline, oxytocin and/or GnRH injection in post-kidding goats on oxytetracyclines residues in goat milk and postpartum ovarian resumption with referring to clinical and haematological pictures

**DOI:** 10.1186/s12917-023-03706-0

**Published:** 2023-09-01

**Authors:** Asem M. Zakaria, Taher Al-Daek, Enas Elmeligy, Ragab H. Mohamed, Eman M. Abu El-Naga, Haitham H. Mohammed, Abdulrahman Abdulkarim, Mohammed Abdelhadi Ali, Khaled. A. Khesruf, Arafat Khalphallah

**Affiliations:** 1 Department of Food Hygiene, Faculty of Veterinary Medicine, Aswan University, Aswan, 81528 Egypt; 2 Faculty of Veterinary Medicine, Omar Al- Mukhtar University, Al-bayda, 919, Al Bayda, Libya; 3 Veterinary Teaching Hospital, Faculty of Veterinary Medicine, Assiut University, Assiut, 71526 Egypt; 4 Department of Theriogenology, Obstetrics, and Artificial Insemination, Faculty of Veterinary Medicine, Aswan University, Aswan, 81528 Egypt; 5 Department of Rangeland, Wildlife and Fisheries Management, Texas A&M University, College Station, TX 77843 USA; 6 Department of Forensic medicine and Toxicology, Faculty of Veterinary Medicine, Assiut University, Assiut, 71526 Egypt; 7 Department of Animal diseases, Faculty of Veterinary Medicine, Aleppo University, Aleppo, Syria; 8 Division of Internal Medicine, Department of Animal Medicine, Faculty of Veterinary Medicine, Assiut University, Assiut, 71526 Egypt

**Keywords:** Clinical findings, Goat, GnRH, Milk oxytetracyclines residues, Oxytocin, Follicles

## Abstract

**Background:**

The post-parturient period in goat had marked changes in an animal’s endocrine and metabolic status as well as by reduction in feed intake when the nutrient demand for impending lactogenesis was increasing. The current study aimed to monitor the residues of oxytetracycline in Baladi goat milk and their hazards on public health as well as the time required until complete disappearance of this medicament from milk through following up periods included 0, 12, 24, 36, 48, 60, 72, 84, 96 and 120 h in post-kidding goat following intrauterine application of oxytetracycline. The study also compared between the efficacy of oxytetracycline only, oxytetracycline with oxytocin, or oxytetracycline with GnRH, through monitoring the clinical findings and haematological pictures at days 0, 5 and 7 post-partum as well as studying the changes in numbers and size of follicles at days 15, 30 and 45 postpartum after different treatments strategies in different groups i.e. Control healthy goat (Cont^gr)^, Oxytetracycline treated goat (Oxytet^gr^), Oxytetracycline-oxytocin treated goat (Oxytet-Oxyto^gr^) and Oxytetracycline-GnRH treated goat (Oxytet-GnRH^gr^). The study was carried out on clinically healthy Baladi goats (n = 40) that gave birth recently. They were divided into 4 equal groups (n = 10 goats for each); Cont^gr^ which received no medication after birth, Oxytet^gr^ which administrated oxytetracycline tablets intrauterine at day of birth, Oxytet-Oxyto^gr^ which treated by oxytetracycline tablets intrauterine at day of birth followed by oxytocin injection at 3rd day after birth, and Oxytet-GnRH^gr^ which treated by oxytetracycline tablets intrauterine at day of birth followed by GNRH injection at 3rd day after birth.

**Results and Conclusions:**

The study concluded the highest oxytetracyclines residues in goats’ milk were reported after 36 h following intrauterine oxytetracycline application where complete disappearance of oxytetracyclines residues in goats’ milk required 120 h elapsed after intrauterine oxytetracycline application in which the goats milk became safe for human consumption. The study also reported powerful influence of the applied variable therapeutic regimens on post-partum ovarian resumption through clear significant variations in numbers and sizes of follicles either between different goats’ groups within the same day, or between days 15, 30 and 45 post-partum within each independent goat group.

## Background

Goats had great ability to adapt and keep themselves in unfavorable environments and induce great alterations in their blood pictures and biochemical indices based on their life physiological phase even if within the same breed. It had characteristic biological, economic, managerial, and social advantages rather than other livestock species and usually termed as the “poor man’s cow.” [1].

Under survival agriculture, goats were reported to be more suitable ruminant animals compared to other ruminants due to their adorable ability to accommodate to harsh environmental conditions. The reproductive ability of goats was the most valuable indicator that estimated durability and continual use as a great resource to progress livelihood [2].

The reproductive condition differed with variable stressors including mainly transition phase of animals. Moreover, the demands of growing fetus and lactogenesis associated with activities of mammary gland postpartum period oftenly caused aggravation of requirement of nutrient during the transitional phases. Such changes needed dramatic physiological and biochemical accommodation during this critical transition period [3].

Accordingly, transition phase considered the most pivotal period in the females’ productive life whereas it had clear effects on their health, fertility, milk yield, and profitability, particularly in high-yielding ones. It was already stated that alterations in different physiological status like breed, age, metabolic disturbances, and reproductive states as well as managemental circumstance including stress due to management, temperature, season, or road transportation had direct effect on hematobiochemical profile in female animal post-partum [4].

Goat milk characterized by its high nutritive value and good digestibility [5]. It had small fat globules, less lactose, easily digested protein and high values of medium and short chain fatty acids such as capric, caprylic, and caproic acids [6]. The smaller fat globules made the goat milk naturally homogenized and provided great surface area for fat to be easily digested by lipase enzyme. Goat milk was recommended for peoples that suffered from buffalo and cow milk allergy also for infants that suffered from lactose intolerance due to its low casein and lactose contents [7].

Clean milk was a milk which obtained from healthy animal and free from chemical, physical and biological hazards. Veterinary drugs were considered as one of the most important chemical hazards that contaminated milk as they found their way to the milk after used in the animal treatment or for prevention of diseases [8].

Residues of antimicrobial drugs in milk had sever public health problems as it might cause drug resistance, direct toxic effect, hypersensitivity, disturbance of normal intestinal flora, skin rashes, teratogenic and carcinogenic effect [9]. Economically, it had a great importance because it caused a great economic loss for dairy products manufacturers because it caused inhibition of starter culture used in the manufacture of fermented dairy products and hence it covered unsanitary condition of milk production [10].

Oxytetracycline considered as one of the broadest spectrum antibiotics that used in animal and human treatment [11]. The intrauterine route of oxytetracycline administration was commonly used for prevention and treatment of endometritis in postpartum dairy animals [12]. It was also used as a prophylactic in goat for estrus synchronization [13]. oxytetracycline was highly effective after parturition in anaerobic environment of the uterus so it was one of the recommended choices for treatment or prevention of endometritis [14]. Following an intrauterine administration of oxytetracycline, it would be concentrated on the endometrium and uterine lumen then it began its action through inducing irritation to endometrium which stimulated inflammatory reaction and uterine defense mechanism followed by infiltration of polymorphonuclear leukocytes cell to uterine lumen [15]. Although intrauterine administration of oxytetracycline was one of the highly recommended choice for endometritis treatment and/or prevention in postpartum dairy animals [16], it could cause drug residues in milk [17]. It was observed that oxytetracyclines residues remained in milk for 1 to 8 days’ post-intrauterine treatment [18].

Accordingly, the objectives of the present work to detect oxytetracycline residues in Baladi goat milk and their hazards on public health as well as the time required for complete their disappearance from milk through following up periods included 0, 12, 24, 36, 48, 60, 72, 84, 96 and 120 h in post-kidding goat following intrauterine application of oxytetracycline. The current work also compared between the therapeutic efficacy of oxytetracycline only, oxytetracycline with oxytocin or oxytetracycline with GnRH through following up the clinical findings and haematological pictures at days 0, 5 and 7 post-partum as well as studying the changes in numbers and size of follicles at days 15, 30 and 45 postpartum following variable applied treatments strategies in different groups i.e. Control healthy goat (Cont^gr)^, Oxytetracycline treated goat (Oxytet^gr^), Oxytetracycline-oxytocin treated goat (Oxytet-Oxyto^gr^) and Oxytetracycline-GnRH treated goat (Oxytet-GnRH^gr^).

## Materials

### Ethics approval and consent to participate

The current study was conducted in accordance with ARRIVE guidelines (https://arriveguidelines.org) and approved by the ethical committee of Faculty of Veterinary Medicine, Assiut University, Egypt licensed number 06/2023/0055 whereas the study is in accordance with the Egyptian bylaws and OIE animal welfare standards for animal care and use in research and education. The authors declared that they obtained informed consent from the owners to use the animals.

### Animals and therapeutic protocol

The experiment study included clinically healthy female Baladi goats recently gave birth (n = 40) with their ages between 1.5 and 2 years. Their body weights ranged between 30 and 40 kg. They were belonged to private farms in Assiut governorates, Egypt. The investigated goat was taken kindly from the farm by a permission that was taken from the farm owner. The investigated goats were divided into 4 groups; control healthy goat (Cont^gr^; n = 10) which received no medication after birth, oxytetracycline treated goat (Oxytet^gr^; n = 10) which received single dose of intrauterine oxytetracycline HC tablets at dose1 tablets/50 kg B.W./12 hrs for 1 days (Terramycin®, each tablet contains 250 mg of oxytetracycline HCl, Pfizer Animal Health, New York, USA) intrauterine at day of birth, oxytetracycline-oxytocin treated goat (Oxytet-Oxyto^gr^; n = 10) which was treated by single dose of intrauterine oxytetracycline HC tablets at dose1 tablets/50 kg B.W./12 hrs for 1 days at day of birth followed by single IM injection of Oxytocin at dose 2 ml (50 IU)/goat (Oxytocin ®, Adwia Co., Cairo, Egypt) at 3rd day after birth, and Oxytetracycline-GnRH treated goat (Oxytet-GnRH^gr^; n = 10) which was treated by single dose of intrauterine oxytetracycline HC tablets at dose1 tablets/50 kg B.W./12 hrs for 1 days at day of birth followed by a single 2ml IM injection of GnRH /goat (Receptal®; 0.004 mg/ml Solution for injection, vial 10 ml, MSD Animal Health, Buckinghamshire, UK) at 3rd day after birth. These animals were subjected to clinical examination and haematological analysis at days 0, 5 and 7 post-partum as well as monitoring of oxytetracyclines residues in milk i.e. At 0-120 h following oxytetracycline application, and changes in number and size of follicles at different post-partum periods at days 15, 30 and 45. The animals were released at the end of the study.

### Samples

whole blood samples that were collected at days 0, 5 and 7 post-kidding on vacutainer tubes with ethylenediamine tetra acetic acid and stored at 4 °C until analysis. All precautions during blood samples collection and preparations were followed as mentioned previously [19].

Milk samples were collected from oxytetracycline treated groups (n = 30; Oxytet^gr^, Oxytet-Oxyto^gr^ and Oxytet-GnRH^gr^) goats were hand milked and milk samples (100 ml each) were collected in a sample tube after 0, 12, 24, 36, 48, 60, 72, 96 and 120 h after intrauterine administration of oxytetracycline tablets which kept frozen (-20 °C) tell be transported to the laboratory to be examined for oxytetracyclines residues by high Performance Liquid Chromatography (HPLC).

### Clinical examination

All goats in the study (n = 40) underwent thorough clinical examination including different parameters mainly temperature, pulse rates, respiratory rates, capillary refill time (CRT) and ruminal motility according to Jackson and Cockcroft [20]; Nagy and Pugh [21].

### Complete blood picture indices

Whole blood picture parameters were manually estimated according to Coles [19]; Harvey [22]; Latimer et al. [23]. They included red blood corpuscles (RBCs), total leucocytic count (TLC), haemoglobin concentrations (Hb) and packed cell volume (PCV).

### HPLC conditions, identification and separation of oxytetracycline in milk samples

#### HPLC conditions

HPLC had a model PU-980 pump, and a SPDM10AVP diode-array detector (Shimadzu Scientific Instruments, Kyoto-Japan), which interfaced with a FMV-5133D7 model personal computer. The analytical column was C18, 250 mm/4.6 mm RP-4 GP non polar sorbent column with a 4% carbon contents. 0.02 M oxalic acid as a mobile-phase, with flow rate 1ml/min. Their detection wave length was 354 nm.

#### Milk sample preparation

The procedure for deproteinizing and extracting of oxytetracyclines in milk was done as follows: 0.2-ml from milk sample and 0.2ml of 10% trichloroacetic acid solution was put into a 1.5 ml micro-centrifuge tube then homogenized using an ultrasonic homogenizer for 20 s. After that the capped tube was centrifuged for 30 min at 4000 rpm. The supernatant fluid was filtered by a disposable syringe filter-unit with a 0.45 μm hydrophilic cellulose**-**acetate membrane. At ambient temperature and flow-rate of 1.0ml/min the filtrate was injected into an HPLC system.

#### Standard preparation

A standard oxytetracycline solution was prepared through weighing 10 mg of oxytetracycline solving in 100ml of 1% (v/v) acetic acid in water solution. These standard solutions were being ready through diluting the stock solution with distilled water and stored in a refrigerator tell be used.

#### Method of validation [identification and separation of oxytetracycline

Identification and separation of oxytetracycline in milk samples depend on its spectrum and retention time which was similar to that of the oxytetracycline prepared standard and its level was measured by the analytical background response depended upon the peak areas in HPLC chromatograms. Each sample required less than 15 min for analysis.

### Number and size of follicles

Number and size of follicles were measured in each group by trans rectal Ultrasonographic examinations at days 15, 30 and 45 post-partum according to Osman et al. [24] using a 6 to 10 MHz linear multifrequency ultrasound transducer (MyLab™One VET, Esaote, Italy).

### Statistical analysis

The estimated data were analyzed using SPSS statistical-software program for windows version 10.0.1 (SPSS Inc., Chicago, IL., USA). The obtained data were expressed as mean ± standard deviation (M ± SD). The data obtained from clinical examination and laboratory analyses as well as milk oxytetracyclines residues monitoring and follicles numbers and sizes estimation, were analysed by general linear model repeated measures ANOVA, and the significance level of results was set at p < 0.05. The significance of differences between the means at different sampling times in each independent goat group either Oxytet^gr^, Oxytet-Oxyto^gr^ or Oxytet-GnRH^gr^, was estimated by Dunnett’s test at p < 0.05. Moreover, the significance of differences between the means in different goat groups i.e. Oxytet^gr^, Oxytet-Oxyto^gr^ and Oxytet-GnRH^gr^, at each separate sampling time, was also estimated by Dunnett’s test at p < 0.05.

## Results

### Clinical findings

Normal clinical manifestations including normal appetite, healthy mucous membranes and capillary refill time (CRT; 1–2 s) were described in all investigated post-kidding goats (n = 40; 100%) in all goat groups through the present work at days 0, 5 and 7 post-partum. These currently examined goats showed no other abnormal findings whereas fever, increased respiratory rates, tachycardia, rumen hypomotility, swollen lymph nodes, lost body weight, diarrhoea and/or melena and abnormal cardiorespiratory signs such as cough, suppurative nasal discharges, abnormal tracheal, heart and lung sounds. Absence of dehydration signs and alopecia was also reported.

The examined post-partum goats throughout the current study showed no remarkable alterations in values of rectal temperatures, pulse, respiratory rates and rumen movements between days 0, 5 and 7 either in Cont^gr^, Oxytet^gr^, Oxytet-Oxyto^gr^ or Oxytet-GnRH^gr^. Moreover, at post-kidding days, no significant variations for these values were also demonstrated between different investigated Baladi goats’ groups either at days 0, 5 or 7 as they were within their reference intervals (Table 1).

**Table 1:** Mean values of temperature, pulse rate, respiratory rate and rumen movements in Cont^gr^ (n = 10), Oxytet^gr^ (n = 10), Oxytet-Oxyto^gr^ (n = 10) and Oxytet-GnRH^gr^ (n = 10) goats

	Cont^gr^			Oxytet^gr^			Oxytet-Oxyto^gr^			Oxytet-GnRH^gr^			Reference values
	Day 0	Day 5	Day 7	Day 0	Day 5	Day 7	Day 0	Day 5	Day 7	Day 0	Day 5	Day 7	
**Temperature** **(°C)**	39.42 ± 0.33^Aa^	39.21 ± 0.62^A1^	39.56 ± 0.71^ A¶^	39.15 ± 0.43^Aa^	39.52 ± 0.47^A1^	39.34 ± 0.24^ A¶^	39.51 ± 0.18^Aa^	39.24 ± 0.22^A1^	39.52 ± 0.61^ A¶^	39.61 ± 0.24^Aa^	39.33 ± 0.51^A1^	39.61 ± 0.15^ A¶^	(39.0–40.5)^20^ or (38–40)^21^
**Pulse** **(Beat/min)**	84.77 ± 2.64^Aa^	83.18 ± 3.07^A1^	84.22 ± 2.34^ A¶^	86.08 ± 3.41^Aa^	84.15 ± 2.51^A1^	84.03 ± 2.33^ A¶^	85.99 ± 2.67^Aa^	84.11 ± 1.88^A1^	86.11 ± 2.16^ A¶^	85.76 ± 1.33^Aa^	86.28 ± 3.67^A1^	84.42 ± 3.17^ A¶^	(70–100)^20^ or (70–90)^21,26^
**Respiration** **(/min)**	23.14 ± 2.35^Aa^	24.01 ± 1.46^A1^	26.21 ± 2.24^ A¶^	25.23 ± 1.88^Aa^	23.72 ± 1.15^A1^	25.22 ± 1.28^ A¶^	24.11 ± 1.26^Aa^	23.88 ± 1.27^A1^	24.17 ± 2.06^ A¶^	24.02 ± 2.11^Aa^	24.44 ± 2.08^A1^	24.38 ± 1.87^ A¶^	(15–30)^20,21^ or (25–35)^26^
**Rumen** **(cycle/2mins)**	3.06 ± 0.64^Aa^	2.88 ± 0.22^A1^	2.97 ± 0.46^ A¶^	2.92 ± 0.76^Aa^	3.03 ± 0.15^A1^	3.01 ± 0.33 ^A¶^	3.01 ± 0.46^Aa^	2.94 ± 0.31^A1^	3.01 ± 0.32^ A¶^	2.88 ± 0.12^Aa^	2.97 ± 0.25^A1^	2.94 ± 0.22^ A¶^	(2–4)^21,26^

Cont^gr^: Control healthy goat. Oxytet^gr^: Oxytetracycline treated goat. Oxytet-Oxyto^gr^: Oxytetracycline-oxytocin treated goat. Oxytet-GnRH^gr^: Oxytetracycline-GnRH treated goat. ^A^Means within the same row with different superscript capital letters between different sampling days either in Cont^gr^, Oxytet^gr^, Oxytet-Oxyto^gr^ or Oxytet-GnRH^gr^, were significantly different. ^a^Means within the same row (At day 0) with different superscript small letters between Cont^gr^, Oxytet^gr^, Oxytet-Oxyto^gr^ and Oxytet-GnRH^gr^, were significantly different. ^1^Means within the same row (At day 5) with different superscript numbers between Cont^gr^, Oxytet^gr^, Oxytet-Oxyto^gr^ and Oxytet-GnRH^gr^, were significantly different. ^¶^Means within the same row (At day 7) with different superscript symbols between Cont^gr^, Oxytet^gr^, Oxytet-Oxyto^gr^ and Oxytet-GnRH^gr^, were significantly different. Reference values according to Jackson and Cockcroft [20]; Nagy and Pugh [21]; Radostits et al. [25]

### Complete blood picture

No significant changes were described in the whole blood picture indices in the post-partum goats throughout the current study whether between days 0, 5 and 7 in each separate independent group, or between the investigated four groups either at days 0, 5 or 7. RBCs, PCV, Hb and PCV values within their reference ranges (Table 2).

**Table 2:** Mean values of whole blood picture indices in Cont^gr^ (n = 10), Oxytet^gr^ (n = 10), Oxytet-Oxyto^gr^ (n = 10) and Oxytet-GnRH^gr^ (n = 10) goats

	Cont^gr^			Oxytet^gr^			Oxytet-Oxyto^gr^			Oxytet-GnRH^gr^			Reference values
	Day 0	Day 5	Day 7	Day 0	Day 5	Day 7	Day 0	Day 5	Day 7	Day 0	Day 5	Day 7	
**RBCs (**_**X**_ **10** ^**12**^ **/L)**	11.86 ± 3.14^Aa^	12.58 ± 2.92^A1^	12.32 ± 2.67^ A¶^	12.44 ± 2.35^Aa^	13.07 ± 3.06^A1^	13.01 ± 2.83^ A¶^	12.11 ± 2.77^Aa^	12.65 ± 2.19^A1^	12.88 ± 2.01^ A¶^	12.41 ± 1.92^Aa^	13.01 ± 2.03^A1^	13.02 ± 1.91^ A¶^	(8–18)^20,31,32^
**PCV** **(L/L)**	0.28 ± 0.02^Aa^	0.30 ± 0.06^A1^	0.31 ± 0.01^ A¶^	0.31 ± 0.03^Aa^	0.28 ± 0.06^A1^	0.32 ± 0.02^ A¶^	0.30 ± 0.01^Aa^	0.32 ± 0.03^A1^	0.34 ± 0.02^ A¶^	0.29 ± 0.02^Aa^	0.31 ± 0.01^A1^	0.30 ± 0.02^ A¶^	(0.22–0.38)^20,31,32^
**Hb** **(g/L)**	106.23 ± 4.97^Aa^	112.07 ± 3.16^A1^	111.34 ± 3.26^ A¶^	109.02 ± 3.85^Aa^	115.13 ± 4.08^A1^	112.37 ± 3.84^ A¶^	108.91 ± 4.13^Aa^	113.27 ± 3.41^A1^	116.74 ± 3.86^ A¶^	110.92 ± 4.06^Aa^	115.17 ± 3.32^A1^	117.08 ± 4.03^ A¶^	(80–120)^20,31,32^
**TLCs (**_**X**_ **10** ^**9**^ **/L)**	8.07 ± 1.01^Ab^	7.88 ± 1.03^A1^	8.01 ± 1.06^ A¶^	7.89 ± 0.84^Aa^	7.96 ± 1.05^B1^	8.14 ± 1.04^B¶^	8.04 ± 1.02^Aa^	8.02 ± 0.93^B1^	8.02 ± 0.81^B¶^	7.96 ± 0.94^Ab^	7.92 ± 0.43^A1^	8.01 ± 1.06^ A¶^	(4–13)^20,31,32^

Cont^gr^: Control healthy goat. Oxytet^gr^: Oxytetracycline treated goat. Oxytet-Oxyto^gr^: Oxytetracycline-oxytocin treated goat. Oxytet-GnRH^gr^: Oxytetracycline-GnRH treated goat. RBCs: Red blood corpuscles. PCV: Packed cell volume. Hb: Haemoglobin concentration. TLCs: Total leukocytic counts. ^A^Means within the same row with different superscript capital letters between different sampling days either in Cont^gr^, Oxytet^gr^, Oxytet-Oxyto^gr^ or Oxytet-GnRH^gr^, were significantly different. ^a^Means within the same row (At day 0) with different superscript small letters between Cont^gr^, Oxytet^gr^, Oxytet-Oxyto^gr^ and Oxytet-GnRH^gr^, were significantly different. ^1^Means within the same row (At day 5) with different superscript numbers between Cont^gr^, Oxytet^gr^, Oxytet-Oxyto^gr^ and Oxytet-GnRH^gr^, were significantly different. ^¶^Means within the same row (At day 7) with different superscript symbols between Cont^gr^, Oxytet^gr^, Oxytet-Oxyto^gr^ and Oxytet-GnRH^gr^, were significantly different. Reference values according to Jackson and Cockcroft [20]; Radostits et al. [26]; Smith [27]

### Oxytetracyclines residues monitoring in goat milk

Mean values of oxytetracyclines residues in goat milk following intrauterine oxytetracycline treatment in oxytetracycline treated goats (n = 30) were significantly (p < 0.05) elevated within the few hours post intrauterine application of oxytetracycline (12–96 h) comparing their values at 0 h (Pre-intrauterine oxytetracycline treatment) whereas oxytetracyclines residues reached their maximum concentrations in goats’ milk at 36 h post-intrauterine oxytetracycline treatment (p < 0.05). Afterwards, these residues were remarkably (p < 0.05) dropped in goat milk at 60–72 h following oxytetracycline treatment until they reached their lowest milk residues’ concentrations (p < 0.05) at 96 h post oxytetracycline (Table 3) treatment comparing with their standards reference values (Table 4). Complete disappearance of oxytetracyclines residues in goat milk were demonstrated at 120 h following intrauterine oxytetracycline treatment (Table 3).

**Table 3:** Mean values of oxytetracyclines residues in goat milk following intrauterine oxytetracycline treatment (ppb = ng/ml) in oxytetracycline treated goats (n = 30)

0 h	12 h	24 h	36 h	48 h	60 h	72 h	96 h	120 h
0^ g^	26.53 ± 8.21^e^	65.49 ± 14.46^c^	119.31 ± 17.32^a^	113.05 ± 15.28^a^	73.52 ± 5.87^b^	39.27 ± 9.54^d^	12.38 ± 4.63^f^	0^ g^

^a−g^Means within the same row with different superscript letters between different sampling hours in oxytetracycline treated goats, were significantly different

**Table 4:** Numbers and percentages of samples exceeding the maximum residues limit (MRL) of oxytetracyclines residues in milk that recommended by different international standards

	12 h		24 h		36 h		48 h		60 h		72 h		96 h		120 h		
	N	%	N	%	N	%	N	%	N	%	N	%	N	%	N	%	
EU (100 ng/ml)	0	0	0	0	6	85.71	5	71.43	0	0	0	0	0	0	0	0	
CODEX (100 ng/ml)	0	0	0	0	6	85.71	5	71.43	0	0	0	0	0	0	0	0	
FDA (300 ng/ml)	0	0	0	0	0	0	0	0	0	0	0	0	0	0	0	0	

EU: European Union. CODEX: Codex Alimentarius Commission. FDA: USA Food and Drug Administration

### Ultrasonographic monitoring of follicles in post-kidding goats

Numbers and sizes for each of medium and large follicles were monitored in the examined goat through the present study using transrectal ultrasonography in all investigated goats groups at different periods post-partum including days 15, 30 and 45. Different measurements were obtained included Total number of follicles/animal (TNF), mean size of large follicles (LFD; ≥5 mm), number of large follicles/animal (NLF), mean size of medium follicles (MFD; 3-5 mm) and number of medium follicles/animal (NMF) in each period in post-kidding goats. ON the other hand, TNF showed no significant changes between the four investigated groups of goats at 15 post-partum days. In contrast, at day 30, TNF had significantly (p < 0.05) higher values in Oxytet-GnRH^gr^ comparing to their values in other three investigated groups. Moreover, these significant variations in TNF values were more pronounced at day 45 post-partum in examined goats whereas TNF values were significantly (p < 0.05) increased at all treated groups comparing with Cont^gr^. These significant changes were also observed between the three treated goats’ groups (Table 5).

**Table 5:** Mean values of numbers and sizes of follicles at 15th, 30th and 45th days postpartum following treatments either in Cont^gr^ (n = 10), Oxytet^gr^ (n = 10), Oxytet-Oxyto^gr^ (n = 10) or Oxytet-GnRH^gr^ (n = 10) goats

	Cont^gr^			Oxytet^gr^			Oxytet-Oxyto^gr^			Oxytet-GnRH^gr^		
	Day 15	Day 30	Day 45	Day 15	Day 30	Day 45	Day 15	Day 30	Day 45	Day 15	Day 30	Day 45
**TNF**	3 ± 0.67^*c^	5 ± 0.79^¶a^	4 ± 0.67$^\checkmark$^b^	3 ± 0.82^*b^	5.2 ± 0.51^¶a^	5 ± 0.82^•a^	3 ± 0.66^*c^	5.3 ± 0.88^¶b^	6 ± 0.67^¶a^	2 ± 0.47^¶c^	6.1 ± 0.79^*b^	7 ± 0.94^*a^
**LFD (≥ 5 mm)**	5.27 ± 0.23^¶b^	5.66 ± 0.46^¶a^	5.71 ± 0.51^¶a^	5.41 ± 0.23^*c^	5.93 ± 0.51^*b^	6.13 ± 0.28^*a^	5.16 ± 0.18^¶b^	5.81 ± 0.29^*a^	5.73 ± 0.16^¶a^	5.42 ± 0.22^*c^	5.73 ± 0.28^*b^	6.21 ± 0.29^*a^
**NLF**	0.7 ± 0.48^*b^	2 ± 0.67^¶a^	2 ± 0.82^¶a^	0.8 ± 0.42^*b^	2.1 ± 0.68^¶a^	2 ± 0.47^¶a^	0.6 ± 0.52^*c^	2.1 ± 0.57^¶b^	3 ± 0.94^*a^	0.8 ± 0.42^*b^	3 ± 0.82^*a^	3 ± 0.82^*a^
**MFD (3-5 mm)**	3.38 ± 0.42^¶b^	4.53 ± 0.26^*a^	4.32 ± 0.34^*a^	3.48 ± 0.23^¶b^	4.43 ± 0.33^*a^	4.53 ± 0.43^*a^	4.03 ± 0.47^*a^	4.03 ± 0.22^¶a^	4.23 ± 0.15^*a^	4.23 ± 0.42^*a^	4.12 ± 0.33^¶a^	4.26 ± 0.27^*a^
**NMF**	2 ± 0.66^*c^	3 ± 0.47^*a^	2 ± 0.67^•b^	2 ± 0.81^*b^	3 ± 0.82^*a^	3 ± 0.81^¶a^	2 ± 0.94^*b^	3 ± 0.67^*a^	3 ± 0.46^¶a^	1 ± 0.68^¶c^	3 ± 0.94^*b^	4 ± 0.82^*a^

Cont^gr^: Control healthy goat. Oxytet^gr^: Oxytetracycline treated goat. Oxytet-Oxyto^gr^: Oxytetracycline-oxytocin treated goat. Oxytet-GnRH^gr^: Oxytetracycline-GnRH treated goat. TNF: Total number of follicles/animal. LFD: Mean size of Large follicles (≥ 5 mm). NLF: Number of large follicles/animal. MFD: Mean size of Medium follicles (3-5 mm). NMF: Number of medium follicles/animal. ^*¶•^$$^\checkmark$$Means within the same row with different superscript symbols between Cont^gr^, Oxytet^gr^, Oxytet-Oxyto^gr^ and Oxytet-GnRH^gr^ either at days 15, 30 or 45, were significantly different. ^abc^Means within the same row with different superscript letters between days 15, 30 and 45 either in Cont^gr^, Oxytet^gr^, Oxytet-Oxyto^gr^ or Oxytet-GnRH^gr^, were significantly different

At days 15 and 45 post-kidding, LFD values were significantly (p < 0.05) higher in Oxytet^gr^ and Oxytet-GnRH^gr^ comparing with their values in other two groups. On the other side, these significant changes in LFD values at day 30 post-partum were observed only between Cont^gr^ and the three treated groups, hence, they were absent between the three treated goats’ groups. LFD values were significantly (p < 0.05) increased the treated goats’ groups when they compared with their values at Cont^gr^. Regarding to NLF values, they showed no significant changes at day 15 post-kidding between all investigated four groups during the present study. In contrast, NLF values were significantly (p < 0.05) elevated particularly in Oxytet-GnRH^gr^ comparing with other goats’ groups at days 30 and 45 post-partum (Table 5).

Oxytet-Oxyto^gr^ and Oxytet-GnRH^gr^ had significantly (p < 0.05) higher values of MFD at day 15 post-partum in comparison with their values in Cont^gr^ and Oxytet^gr^. In contrast, Cont^gr^ and Oxytet^gr^ had significantly (p < 0.05) higher values of MFD at day 30 post-partum comparing with their values in Oxytet-Oxyto^gr^ and Oxytet-GnRH^gr^. At day 45 post-partum, these significant variations in MFD values were not reported between all different goats’ groups (Table 5).

NMF values had remarkable changes at days 15 and 45 following parturition between treated goats’ groups particularly Oxytet-GnRH^gr^, and Cont^gr^ meanwhile these remarkable changes were not stated at day 30 post-partum between all investigated goats’ groups. At day 15, NMF values were significantly (p < 0.05) dropped in Oxytet-GnRH^gr^ comparing with those in other investigated groups, while these changes were absent between other three investigated groups. In contrast, at day 45 post-kidding, NMF values were remarkably (p < 0.05) higher in Oxytet-GnRH^gr^ comparing with those in the other three investigated groups. Moreover, Oxytet^gr^ and Oxytet-Oxyto^gr^ had significantly (p < 0.05) higher values of NMF comparing with those in Cont^gr^ (Table 5).

TNF, LFD and NLF values were significantly (p < 0.05) higher at days 30 and 45 than those at day 15 either in Cont^gr^, Oxytet^gr^, Oxytet-Oxyto^gr^, or in Oxytet-GnRH^gr^. MFD values were significantly (p < 0.05) elevated at days 30 and 45 when they compared with their values at day 15 either in Cont^gr^ or in Oxytetgr. In contrast, these significant changes of MFD values were not reported between days 15, 30 and 45 post-partum either in Oxytet-Oxyto^gr^ or in Oxytet-GnRH^gr^. NMF values had significant variations between days 15, 30 and 45 either in Cont^gr^, Oxytet^gr^, Oxytet-Oxyto^gr^, or in Oxytet-GnRH^gr^ whereas they were significantly (p < 0.05) higher at days 30 and 45 comparing with those at day 15 either in Oxytet^gr^, Oxytet-Oxyto^gr^, or in Oxytet-GnRH^gr^, meanwhile NMF values were significantly (p < 0.05) higher at day 30 comparing with those at days 15 and 45 in Cont^gr^ (Table 5).

## Discussion

### Clinical findings

Goats had a puerperal anoestrus during the postpartum phase due to physiological alterations in their reproductive tract to induce a new conception. Anoestrus occurred as a consequence of uterine tissue renewal i.e. uterine involution, that was related with the return of cyclic ovarian activities, and was clearly affected by several factors like the body condition score before and after birth, social interactions, period of lactation, suckling of the offspring, intensity of negative energy balance and stress from adverse climatic conditions i.e. the heat [28]. Regrading to the present study, normal clinical manifestations including normal appetite, healthy mucous membranes and capillary refill time (CRT; 1–2 s) were described in all investigated post-kidding goats (n = 40; 100%) through the present work at days 0, 5 and 7 post-partum. No other abnormal findings were described. Furthermore, the examined post-partum goats throughout the current study showed no remarkable alterations in rectal temperatures, pulse, respiratory rates and rumen movements between days 0, 5 and 7 in each independent investigated group. Moreover, at post-kidding days, no significant variations for these values were also demonstrated between the four investigated goats’ groups either at days 0, 5 or 7 as they were within their reference intervals reported by Jackson and Cockcroft [20]; Nagy and Pugh [21]; Radostits et al. [25]. On other side, previous reports mentioned significant changes in endocrine and metabolic conditions in post-parturient animals as well as a reduction in feed intake when the nutrient requirements for impending lactogenesis was aggravated [1].

### Complete blood picture

Hematological parameters were strong indicators of physiological healthy conditions as their evaluation was necessary in monitoring the animal response to different physiological stressful status including pregnancy and parturition as well as lactation [1, 29, 30]. In general, red blood cell indices decreased during pregnancy and remained low for a few weeks’ post-partum in ewes, cows, sows, mares, and bitches [31]. However, great alterations in hematological indicators existed between different breeds of goat [32]. The current work revealed no significant changes in the whole blood picture indices following kidding in goats under experiments whether between days 0, 5 and 7 in each independent goat group, or between all investigated four goats’ groups either at days 0, 5 or 7. RBCs, PCV, Hb and PCV values within their reference ranges reported by Jackson and Cockcroft [20]; Radostits et al. [26]; Smith [27]. On the other side, hematobiochemical parameters were characteristic of health status in post-kidding goats. Although the estimated hematological parameters were within normal reference intervals, remarkably lower levels of PCV, Hb, and TLC in goats during first 2 weeks post-kidding when compared to the control one were mainly indicative of stress [1]. A significantly lower Hb values was reported on the day of kidding then they were increased significantly up to day 45 of lactation. An elevation of Hb values during the post-partum period might be owned to higher oxygen demands and greater metabolic rates requirements [33, 34]. Non-significant differences in Hb concentrations were demonstrated on 0 day and 7 days post-kidding [35] in Malabari goats, − 3 to + 3 weeks of kidding [3]. Regarding to PCV values, the current results contradicted with the previous reports which reported an increasing in PCV values from 0 to 45 days in post-partum goats. Reduction in PCV concentrations on the day of parturition had also been mentioned by Tharwat et al. [3]. This drop in PCV levels on the parturition day was attributed to stress associated with parturition [36]. Furthermore, the reduction in PCV values on the day of parturition might be due to hemodilution effect emitted from an elevation in plasma volume or raise water mobilization to mammary glands tissues through the vascular system [37]. In contrast with the results of TLC in the current study in post-kidding goats, Manat et al. [1] reported significantly lower TLC values reported on 0 day while TLC values differed non-significantly on 7th to 45th day post-partum. Moreover, lowest values of TLC were demonstrated on the day of kidding that might be attributable to stress or drop in immunity.

### Oxytetracyclines residues monitoring in goat milk

According to the latest regulations for the antimicrobials use in animal treatment, that were introduced in 2022, many antibiotics were among the restricted agents, while oxytetracycline remained among the antimicrobial with alternatives in veterinary and human medicine due to their indications as well as their low risk for the spread of drug resistance [38, 39]. Therefore, the prudent reasonable use of oxytetracycline, based on a perfect knowledge of pharmacokinetics and the latest information on the bacterial sensitivity was necessary and critical for clinical therapeutic efficacy. Many articles studied the pharmacokinetics of oxytetracyclines following their intrauterine application as well as its spread in blood and milk [40, 41]. Due to presence of drug residues in meat and milk after animal treatment, Food and Drug Administration (FDA) needed an accurate method for of detecting and quantifying drug residues to achieve the safety of the food supply. HPLC was accurate, sensitive and rapid method for detection of antimicrobial or drugs residues in milk [41]. In the present study, HPLC was used to measure the level of oxytetracyclines residues in goat milk following intrauterine administration. Values of oxytetracyclines residues in goat milk following intrauterine oxytetracycline treatment in oxytetracycline treated goats (n = 30) were significantly elevated within the few hours post intrauterine application of oxytetracycline (12–96 h) comparing their values at 0 h (Pre-intrauterine oxytetracycline treatment) whereas oxytetracyclines residues reached their maximum concentrations in goats’ milk at 36 h post-intrauterine oxytetracycline treatment. Afterwards, these residues were remarkably dropped in goat milk at 60–72 h following oxytetracycline treatment until they reached their lowest milk residues’ concentrations at 96 h post oxytetracycline treatment comparing with their standards reference values at Table 4. Complete disappearance of oxytetracyclines residues in goat milk were demonstrated at 120 h following intrauterine oxytetracycline treatment. On other hand, Tan et al. [18] said that oxytetracyclines residues remained in milk for 1 to 8 days after intrauterine treatment while Romero et al. [13] detected oxytetracycline in goat milk at day four after intrauterine treatment. Makki et al. [14] detected oxytetracycline in cow milk at 70 h after intrauterine treatment with maximum concentration at 22 h. Moreover, other articles also added that since oxytetracycline left residues in milk used for human consumption, which could cause allergy reactions in hypersensitive persons, and spread of drugs-resistant microorganisms [42]. European Union (EU) [43], Codex Alimentarius Commission (CAC) [44] and USA FDA [45] had established a maximum residual limit (MRL) of oxytetracycline in milk at 100ng/ml, 100 ng/ml, and 300 ng/ml, respectively. At the same time FDA recommended acceptable daily intake (ADI) 0–30 ng/ml to avoid any harmful hazardous health effects on consumers [46]. Data reported in Table (4) indicated that oxytetracyclines residues level in milk reached the MRL set by EU and CODEX at 24 and 36 h after intrauterine treatment and the milk became safe for human consumption after 96 h from the end of intrauterine treatment.

### Ultrasonographic monitoring of follicles in post-partum goats

Many hormonal programs were applied to stimulate estrus, as well as the use of melatonin implants, artificial light, and male-effect [47]. To achieve perfect estrous synchronization, progesterone (P4) (or analogues) was commonly used [48]. The current work used different therapeutic protocols to induce ovulation in goats whereas numbers and sizes for each of medium and large follicles were monitored in the examined goat subjected to variable therapeutic programs through the present study using transrectal ultrasonography in all investigated goats’ groups at different periods post-partum including days 15, 30 and 45. Different measurements were obtained included TNF, LFD (≥ 5 mm), NLF, MFD (3-5 mm) and NMF in each period in post-partum goats. On other hand, Pietroski et al. (2013) used different protocols depended on using progestagen sponges for different times included either 6, 9 or 12 days were equally effective to produce a synchronized estrus during the non-breeding season in investigated Saanen goats. The identification of ovulation in association with the onset of estrous might be different regarding to the selected treatment whereas this moment was very necessary to establish efficacious systems or strategies of artificial insemination. These new probabilities of estrous induction-synchronization protocols were attractive as they allowed producers to provide dairy products and meat throughout the year. Regarding to TNF in the current study, TNF showed no significant changes between the treated goats groups at 15 post-partum days. In contrast, at day 30, TNF had significantly higher values in Oxytet-GnRH^gr^ comparing to their values in the other examined goats’ groups. Moreover, these significant variations in TNF values were more pronounced at day 45 post-partum in examined goats whereas they were significantly higher in the treated goats’ groups than those in Cont^gr^. They were also significantly higher at Oxytet-GnRH^gr^ comparing with the other three investigated groups. On other hand, referring to LFD values at days 15 and 45 post-kidding, they were significantly higher in Oxytet^gr^ and Oxytet-GnRH^gr^ comparing with their values in Cont^gr^ and Oxytet-Oxyto^gr^. On the other side, these significant changes in LFD values at day 30 post-partum were observed between Cont^gr^ and treated groups of goats, hence, they were absent between the treated goats’ groups. LFD values were significantly increased the three treated groups when they compared with their values at Cont^gr^. In contrast, the previous literature mentioned a reduction in the frequency of GnRH pulses over the anestrous period that might be owned to low values of circulating serum estradiol. A follicular growth had been reported during this period as these follicles were reacting to infrequent LH pulses due to the estradiol secretion. The emergence of a follicular wave might be attributable to fluctuations in plasma FSH values. The follicles reached their pre-ovulatory sizes but went into atresia [49, 50]. Through the current work, NLF values showed no significant changes at day 15 post-kidding between the four investigated goats groups. In contrast, NLF values were significantly elevated particularly in Oxytet-GnRH^gr^ comparing with other goats’ groups at days 30 and 45 post-partum. Moreover, Oxytet-Oxyto^gr^ and Oxytet-GnRH^gr^ had significantly higher values of MFD at day 15 post-partum in comparison with their values in Cont^gr^ and Oxytet^gr^. In contrast, Cont^gr^ and Oxytet^gr^ had significantly higher values of MFD at day 30 post-partum comparing with their values in Oxytet-Oxyto^gr^ and Oxytet-GnRH^gr^. At day 45 post-partum, these significant variations in MFD values were not reported between all investigated goats’ groups. On the other side, the previous report using other medicaments regarding reproduction in ruminants mainly goats mentioned that the numbers (1.6) of ovulations and diameters of the largest follicle (5.7 mm) were not variable among variable treatments. It was well established that blood P4 levels might affect luteinizing hormone (LH) and so long-course treatments could cause subluteal values of blood p4, inducing an excessive growth as well as persistence of the largest follicle prolonging its age [51]. This result was not reported by Pietroski et al. [52] that might be attributed to the used long-term applicable treatment in their study was not as prolonged as in other experiments. A total of 83% of female goats ovulated; similar to the 96% previously reported [53]. One female goat ovulated before detecting estrus manifestations that might be induced by functions of dominant-subordinate relationships [52, 54]. The current study reported remarkable changes in NMF values at days 15 and 45 post-kidding between treated goats’ groups mainly Oxytet-GnRH^gr^, and control goats meanwhile these remarkable changes for NMF values were not stated at day 30 post-partum between all investigated goats’ groups. At day 15, NMF values were significantly dropped in Oxytet-GnRH^gr^ comparing with those in other investigated goats group, while these changes were absent between Cont^gr^, Oxytet^gr^ and Oxytet-Oxyto^gr^. In contrast, at day 45 post-kidding, NMF values were remarkably higher in Oxytet-GnRH^gr^ comparing with those other investigated goats’ groups. Moreover, these significant elevations in NMF values were reported in all treated groups comparing with Cont^gr^. The previous report confirmed that most dairy does were single-fetal mammals, with 1 to 3 mature follicles per estrus cycle, while other follicles of these dairy goats were inhibited by apoptosis and atresia. Based on theory of classic reproductive physiology in goats, the formation and development of these mature follicles as well as the ovulation occurrence commonly depended on the combined effects of LH and follicle stimulating hormone (FSH). Furthermore, selection of dominant follicles selection was mainly depended on two aspects: the first was the blood gonadotropin values, while the other aspect was the hormone receptors expression in these follicles [55, 56].

The present study reported significant changes in numbers and sizes of follicles between days 15, 30 and 45 post-kidding in examined goats whereas TNF, LFD and NLF values were significantly higher at days 30 and 45 than those at day 15 in each independent group of the investigated goats. MFD values were significantly elevated at days 30 and 45 when they compared with their values at day 15 either in Cont^gr^ or in Oxytetgr. In contrast, these significant changes of MFD values were not reported between days 15, 30 and 45 post-partum in each independent group of the investigated goats. NMF values had significant variations between days 15, 30 and 45 either in Cont^gr^, Oxytet^gr^, Oxytet-Oxyto^gr^, or in Oxytet-GnRH^gr^ whereas they were significantly higher at days 30 and 45 comparing with those at day 15 in each independent group of the investigated goats, meanwhile NMF values were significantly higher at day 30 comparing with those at days 15 and 45 in Cont^gr^. On the other hand, as stated by Pietroski et al. [52], human chorionic gonadotropin (hCG) was administered 5 days following breeding to evade fetal loss because it was reported as a powerful luteotropic or anti-luteolytic agent in goat [57]. However, 21% of goats (4/19) lost their fetuses, which was still a higher percentage. It was earlier proposed that the hCG action appeared to depend on parity and season [58]. Finally, Nogueira et al. [59] added that Goats supplied with maize in their rations provided higher metabolizable energy at a level of 1 or 1.5 times maintenance greatly elevated rates of ovulation in comparison with those fed a diet free from maize but supplied metabolizable energy at the level of one times maintenance. This explained the excellent sensitivity of the ovary in goats to changes in dietary constituents and clarified the fact that fertility could greatly be affected by moderate changes in the diet and within a relatively short period of maize supplementation.

## Conclusion

The study concluded the highest oxytetracyclines residues in goats’ milk were reported after 36 h following intrauterine oxytetracycline application where complete disappearance of oxytetracyclines residues in goats’ milk required 120 h elapsed after intrauterine oxytetracycline application in which the goats milk became safe for human consumption. The study also reported powerful influence of the applied variable therapeutic regimens on post-partum ovarian resumption through clear significant variations in numbers and sizes of follicles either between days 15, 30 and 45 post-partum within the same goat group, or between different goats’ groups within the same day i.e. days 15, 30 or 45.

## Data Availability

The datasets used and/or analyzed during the current study are available from the corresponding author on reasonable request. Data was also available after publishing in this journal.

## Abbreviations

CAC: Codex Alimentarius Commission
Cont^gr^: Control healthy goat
CRT: Capillary refill time
EU: European Union
FDA: USA Food and Drug Administration
FSH: Follicle stimulating hormone
Hb: Haemoglobin
hCG: Human chorionic gonadotropin
HPLC: High Performance Liquid Chromatography
LFD: Mean size of Large follicles (≥ 5mm)
LH: Luteinizing hormone
MFD: Mean size of medium follicles (3-5mm)
NLF: Number of large follicles/animal
NMF: Number of medium follicles/animal
Oxytet^gr^: Oxytetracycline treated goat
Oxytet-GnRH^gr^: Oxytetracycline-GnRH treated goat
Oxytet-Oxyto^gr^: Oxytetracycline-oxytocin treated goat
PCV: Packed cell volume
P4: Progesterone
RBCs: Red blood corpuscles
TLC: Total leucocytic count
TNF: Total number of follicles/animal
